# Comparative chemical genomics reveal that the spiroindolone antimalarial KAE609 (Cipargamin) is a P-type ATPase inhibitor

**DOI:** 10.1038/srep27806

**Published:** 2016-06-13

**Authors:** Gregory M. Goldgof, Jacob D. Durrant, Sabine Ottilie, Edgar Vigil, Kenneth E. Allen, Felicia Gunawan, Maxim Kostylev, Kiersten A. Henderson, Jennifer Yang, Jake Schenken, Gregory M. LaMonte, Micah J. Manary, Ayako Murao, Marie Nachon, Rebecca Murray, Maximo Prescott, Case W. McNamara, Carolyn W. Slayman, Rommie E. Amaro, Yo Suzuki, Elizabeth A. Winzeler

**Affiliations:** 1 Division of Pharmacology and Drug Discovery, Department of Pediatrics, University of California, San Diego, School of Medicine, La Jolla, California, USA; 2 Department of Synthetic Biology and Bioenergy, J. Craig Venter Institute, La Jolla, California, USA; 3 Department of Chemistry & Biochemistry and the National Biomedical Computation Resource, University of California, San Diego, La Jolla, California, USA; 4 Department of Genetics, Yale University School of Medicine, New Haven, Connecticut, USA; 5 Basic Sciences Division, Fred Hutchinson Cancer Research Center, Seattle, WA, USA; 6 Genomics Institute of the Novartis Research Foundation, San Diego, California, USA

## Abstract

The spiroindolones, a new class of antimalarial medicines discovered in a cellular screen, are rendered less active by mutations in a parasite P-type ATPase, *PfATP4*. We show here that *S. cerevisiae* also acquires mutations in a gene encoding a P-type ATPase (*ScPMA1*) after exposure to spiroindolones and that these mutations are sufficient for resistance. KAE609 resistance mutations in *ScPMA1* do not confer resistance to unrelated antimicrobials, but do confer cross sensitivity to the alkyl-lysophospholipid edelfosine, which is known to displace *Sc*Pma1p from the plasma membrane. Using an *in vitro* cell-free assay, we demonstrate that KAE609 directly inhibits *Sc*Pma1p ATPase activity. KAE609 also increases cytoplasmic hydrogen ion concentrations in yeast cells. Computer docking into a *Sc*Pma1p homology model identifies a binding mode that supports genetic resistance determinants and *in vitro* experimental structure-activity relationships in both *P. falciparum* and *S. cerevisiae*. This model also suggests a shared binding site with the dihydroisoquinolones antimalarials. Our data support a model in which KAE609 exerts its antimalarial activity by directly interfering with P-type ATPase activity.

The spiroindolones, a novel class of orally bioavailable antimalarials discovered in a phenotypic whole-cell screen, have been shown in Phase II clinical trials to rapidly clear parasites from adults with uncomplicated *P. vivax* and *P. falciparum* malaria^1^. KAE609 (cipargamin or NITD609), a representative compound, works twice as fast in patients as the current gold standard treatment, artemisinin. KAE609 possesses both the potency (average IC_50_ of 550 pM against asexual blood-stage *P. falciparum*)^2^ and favorable pharmacokinetics (elimination half-life of ~24 hours in humans)^3^ needed for a single-dose cure, a feature that could help slow the onset of parasite resistance and that is not shared by existing, approved antimalarial drugs. KAE609 is also unique in its ability to block transmission to mosquitoes^4^.

Despite promising activity in both cellular and organismal assays, the spiroindolones act by a relatively uncharacterized mechanism. Directed-evolution experiments in parasites have shown that resistance is conferred by mutations in the gene encoding the parasite plasma membrane P-type ATPase, *Pf*Atp4p. Biophysical studies have shown that parasites treated with KAE609 are not only unable to extrude intracellular sodium, but also exhibit changes in intracellular pH^5^. On the other hand, *PfATP4* mutations also confer resistance to a variety of unrelated chemical scaffolds with antimalarial activity^6^,^7^,^8^,^9^, suggesting that *PfATP4* may be a multidrug resistance gene instead of the true spirondolone target.

Although direct work on *Pf*Atp4p would be desirable, our attempts to study recombinant *Pf*Atp4p have not been successful (our unpublished data) nor is a structure available. To better understand the mechanism of action of spiroindolones, we used directed evolution experiments in baker’s yeast (*Saccharomyces cerevisiae*), a more genetically tractable model system.

## Results

### KAE609 inhibits *S. cerevisiae* growth

To determine whether yeast could be used to study the function of KAE609, we tested the compound in a cellular, phenotypic assay. Using yeast proliferation as a readout (OD600), we found the half maximal inhibitory concentration (IC_50_) of KAE609 against a wild-type strain (SY025) to be prohibitively high for drug-selection studies (IC_50_ = 89.4 ± 18.1 μM, 9 observations). Reasoning that the yeast cells might be expelling KAE609 via drug efflux pumps, we next tested a strain that lacks 16 genes encoding ATP-binding cassette (ABC) transporters, termed “ABC_16_-Monster”^10^. As predicted, KAE609 was more potent against ABC_16_-Monster (IC_50_ = 6.09 ± 0.74 μM), suggesting that this yeast strain could be a useful surrogate for malaria parasites.

### KAE609 resistance is conferred by mutations in *ScPMA1*, an ortholog of *PfATP4*

**Figure 1:**
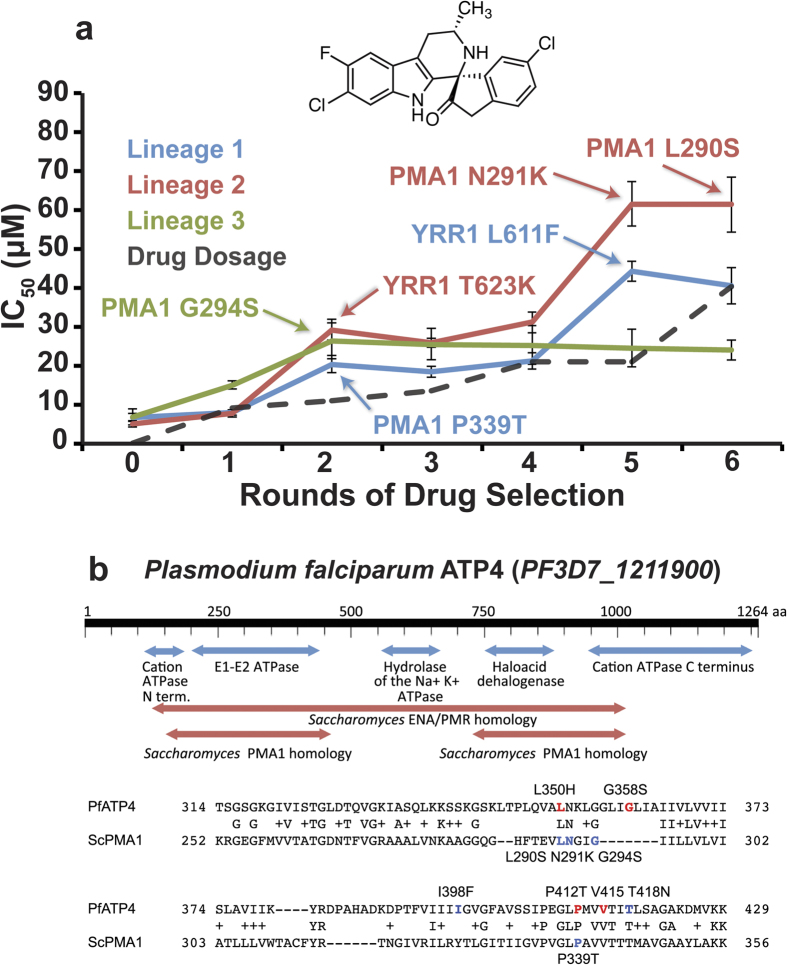
KAE609 directed evolution produces mutations in *ScPMA1*. (**a**) An IC_50_ analysis of ABC_16_-Monster resistant lines showed that resistance developed over multiple rounds of drug selection. Sanger sequencing of *ScPMA1* and *ScYRR1* at each round was used to determine when each mutation (highlighted) arose in its respective lineage. (**b**) Alignment of *Pf*Atp4p to *Sc*Pma1p (S288c) using PSI-BLAST (Position-Specific Iterated BLAST)^68^. The figure shows conserved domains (*Pf*Atp4p amino acid 139–462:*Sc*PMA1 87–389; *Pf*ATP4 744–1032:*Sc*PMA1 527–784). *Sc*Pma1p and *Pf*Atp4p are homologous only in the E1-E2 ATPase domain. The alignments of *Pf*Atp4p amino acids 314–429 and *Sc*Pma1p 252–356 are shown below. Residues that confer resistance in *Plasmodium* when mutated are colored based on the compound class used: red for dihydroisoquinolones and blue for spiroindolones (see^38^ for a review).

We next sought the KAE609 target in *S. cerevisiae* using the same *in vitro* evolution and whole-genome scanning method that previously identified *PfATP4* as a KAE609 resistance gene^2^. ABC_16_-Monster cells were exposed to increasing KAE609 concentrations in three clonal cultures. In all three cultures, compound resistance emerged after two rounds of selection, with new IC_50_ values of 20.4 ± 2.2, 29.1 ± 2.6, and 26.4 ± 4.6 μM, respectively. After an additional three rounds of selection, two of the cultures developed additional resistance (40.5 ± 4.7 and 61.5 ± 7.1 μM) (Fig. 1a). To determine the genetic basis of this *in vitro* resistance, we prepared genomic DNA from clonal strains of the terminal selection. Samples were fragmented, labeled, and sequenced with >40-fold coverage (Supplemental Table 1). The sequences were then compared to the sequence of the parental clone.

Sequencing revealed 5–8 single nucleotide variants (SNVs) in each line and no additional copy number variants (CNVs) beyond the 16 ABC_16_-transporter deletions and selection-marker insertions characteristic of the strain. Among the SNVs, there were 2–3 missense mutations in protein-coding genes per clone (Table 1). The transcription factor *ScYRR1* was mutated in two lineages. *ScPMA1* was the only gene mutated in all three clones. *ScPMA1*, a gene that encodes a P-type ATPase responsible for maintaining hydrogen-ion homeostasis across the plasma membrane in yeast^11^, was the only essential gene among those identified. It is also a homolog of a KAE609 resistance-conferring gene in *P. falciparum*, *PfATP4* (Fig. 1b). The identified *ScPMA1* mutations (Pro339Thr, Leu290Ser, and Gly294Ser) are clustered in the E1-E2 ATPase domain, in a region that is homologous to *PfATP4.* These mutated amino acids are positioned near or at the same homologous residues that confer parasite resistance to both the spiroindolones and the dihydroisoquinolones, another compound class predicted to inhibit *Pf*Atp4p^7^. Sanger sequencing of *ScPMA1* and *ScYRR1* at each round of selection was used to determine when each mutation arose in its respective lineage. This same sequencing also identified an additional clone in Lineage 2 with its own distinct *ScPMA1* mutation (Asn291Lys). Mutations in *ScPMA1* and *ScYRR1* each correlate with increased KAE609 resistance (Fig. 1a).

### *ScPMA1* alleles are sufficient to confer resistance to KAE609

To further investigate the contribution of different alleles to the resistance phenotypes, we determined whether the mutations we found were specific to the spiroindolones. We performed 103 additional directed-evolution experiments in ABC_16_-Monster against 26 different compounds with blood-stage *P. falciparum* activity. None of the 103 genomes sequenced had *ScPMA1* mutations. However, 22 clones resistant to six unrelated compounds also had *ScYRR1* mutations (Supplemental Table 2). These findings suggest that *ScPMA1* is the spiroindolone target, and *ScYRR1* is a more general resistance gene.

**Figure 2:**
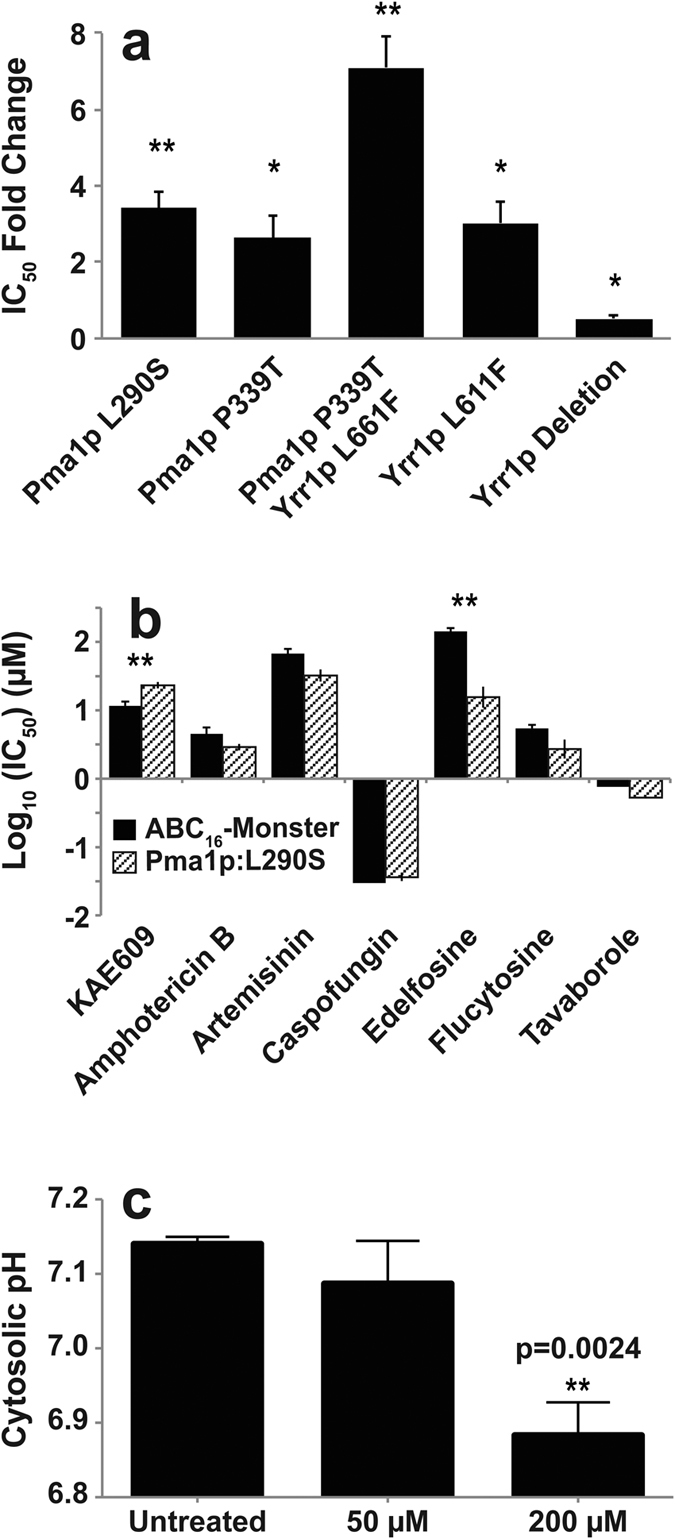
Mutations in *ScPMA1* confer resistance to KAE609, sensitize yeast to edelfosine, and treatment with KAE609 leads to a change in cytosolic pH. (**a**) Changes in ABC_16_-Monster KAE609 resistance associated with mutations introduced using the CRISPR/Cas9 system, relative to the unmodified parent line. (**b**) The CRISPR mutant containing the *Sc*Pma1p:L290S amino acid change was tested for cross resistance or sensitivity to a range of antimicrobials. It shows 7.5-fold cross sensitivity to edelfosine, a chemical known to indirectly inhibit *Sc*Pma1p function. (**c**) The effect of KAE609 treatment on cytosolic pH was measured using cytosolically expressed ratiometric pHluorin, a pH-sensitive GFP^19^. Cytosolic pH was measured for three biological replicates. pH values are represented as mean ± standard deviations. P values for IC_50_ fold-changes were determined using a one-tailed ratio paired t-test comparing the ratio of the mutant-strain IC_50_ value to that of the parental strain. For all bar graphs, (*) indicates *p* < 0.05 and (**) indicates *p* < 0.01).

To separate out the individual alleles’ contribution to resistance, genetic validation using the CRISPR/Cas system was performed. These experiments confirmed that mutations in *ScPMA1* and *ScYRR1* both cause a 2.5 fold increase in KAE609 resistance and that they have a multiplicative effect, as observed in the directed-evolution experiments. However, *Sc*Yrr1p does not appear to be the primary KAE609 target. When we deleted *ScYRR1*, the resulting strain was viable, demonstrating that *ScYRR1* is not essential. Furthermore, KAE609 potency increased in the deletion mutant, further suggesting that *Sc*Yrr1p confers resistance through an indirect mechanism (e.g., by activating the transcription of proteins that can detoxify KAE609, Fig. 2a).

### ScPMA1 mutation confers sensitivity to edelfosine, but not other antimicrobials

Having shown that *ScPMA1* mutations are sufficient to generate KAE609 resistance, we next sought to determine the specificity of the resistance. We tested the L290S CRISPR-engineered *ScPMA1* mutant against a set of known antimicrobials with mechanisms unrelated to *Sc*Pma1p. We also included the alkyl-lysophospholipid edelfosine as a positive control because it reduces *Sc*Pma1p plasma-membrane concentrations by selectively displacing *Sc*Pma1p for endosomal degradation^12^,^13^,^14^,^15^. None of the unrelated antimicrobials demonstrated cross-resistance or sensitivity in the *ScPMA1* mutant. However, the mutant did demonstrate 7.5-fold increase in sensitivity to edelfosine, suggesting that the mutation confers a fitness cost to the functioning of *Sc*Pma1p (Fig. 2b).

### KAE609 exposure increases intracellular acidity in yeast

Although homology modeling and functional evidence indicates that PfAtp4p maintains sodium homeostasis^5^, *Sc*Pma1p functions as the major proton pump in *Saccharomyces* and is essential for cell viability^16^. It is responsible for maintaining pH homeostasis by pumping protons out of the cell; disruption of *Sc*Pma1p function produces a drop in intracellular pH as hydrogen ions accumulate in the cytosol^17^,^18^. If KAE609 inhibits *Sc*Pma1p, we would expect a drop in cytosolic pH after drug exposure. Therefore, intracellular pH was measured using a strain of *S. cerevisiae* expressing the pH-sensitive green fluorescent protein (pHluorin)^19^. Since these cells are not ABC_16_-transporter multi-knockouts, higher KAE609 dosages were required. After 3 hours of treatment with 200 μM KAE609, the cytoplasmic pH dropped from 7.14 ± 0.01 to 6.88 ± 0.04. This equates to an 80.6% increase in the cytoplasmic hydrogen ion concentration (p = 0.0024) (Fig. 2c), consistent with the hypothesis that KAE609 prevents *Sc*Pma1p from pumping hydrogen ions from the cytoplasm to the extracellular space.

### KAE609 homology model explains mutations

**Figure 3:**
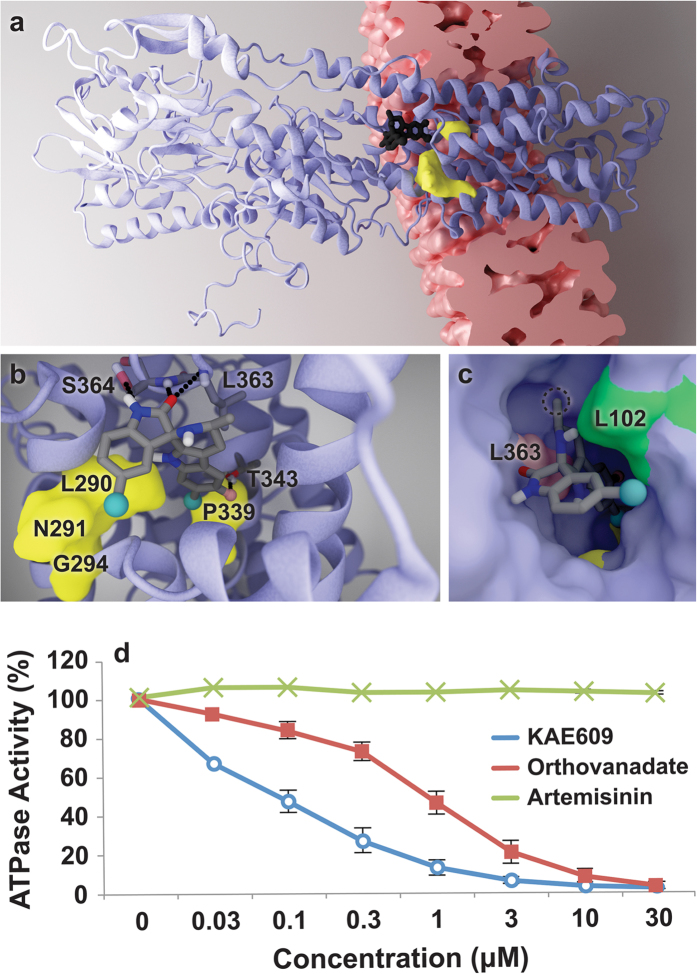
Functional and docking analyses support the hypothesis that KAE609 is a direct *Pf*Atp4p inhibitor. (**a–c**) Illustrations of the *Sc*Pma1p homology model and KAE609 docked pose. (**a**) The docked ligand is shown in black. Amino acids associated with evolved resistance are shown in yellow. The lipid-bilayer location was predicted using PDBID 1MHS^69^, CHARMM-GUI^70^, and the OPM database^71^. (**b**) The ligand with predicted hydrogen-bond partners (Ser364, Thr343, and Leu363). (**c**) The receptor in surface representation. Leu102 (green) is predicted to form hydrophobic contacts with KAE609. The position of Leu363 (pink) explains why the chirality at the 3′ carbon atom is critical to potency; if inverted, the attached methyl group (circled) would clash sterically with Leu363. (**d**) KAE609 inhibition of *Sc*PMA1p in the vesicle-based assay^25^. Orthovanadate, a non-specific ATPase inhibitor, and artemisinin, an unrelated antimalarial, are included as controls. Two independent experiments were performed.

To investigate KAE609 binding in more detail, we created a homology model of wild-type *Sc*Pma1p (UniProt ID: P05030) in the E2 (cation-free) state and mapped the four identified mutations (Leu290Ser, Pro339Thr, Gly294Ser, and Asn291Lys) onto the protein. The altered amino acids line a well-defined, cytoplasm-accessible pocket within the membrane-spanning domain that is large enough to accommodate a small molecule (Fig. 3). The computer-docking program Glide XP^20^,^21^,^22^ was next used to position KAE609 within the predicted wild-type pocket, with minimal manual adjustments. The docked pose placed the tricyclic tetrahydropyridoindole (THPI) moiety of the ligand between two residues that were mutated during directed evolution (Leu290 and Pro339, Fig. 3a,b), providing a plausible explanation for why these residues are so critical for KAE609 binding.

The docked pose suggests that receptor-ligand interactions are governed predominantly by high shape complementarity and hydrophobic contacts, provided by residues such as Leu102 (Fig. 3b,c). That both the Leu290Ser and Pro339Thr mutations substituted nonpolar with polar amino acids further supports the hypothesis that hydrophobicity plays a critical role. We note that Pro339Thr in *Sc*PMA1p corresponds to Pro415Thr in *Pf*Atp4p. In malaria parasites, the latter amino-acid modification is known to confer resistance to the dihydroisoquinolones, another class of antimalarials believed to target *Pf*Atp4p by binding into the same pocket that we here identify as the KAE609 binding site^7^. Interestingly, our data showed that Leu290Ser ABC_16_-Monster yeast were sensitized to the action of (+)-SJ000571311 (also known as SJ311), a representative dihydroisoquinolone^7^ resulting in a small shift from 133.4 ± 23.4 to 83.65 ± 7.98 uM (Supplemental Table 3).

KAE609 may also form multiple hydrogen bonds with Ser364 and Leu363. The backbone amino groups of both these residues are predicted to form hydrogen bonds with the Ser364 ketone oxygen atom at the 2 position. Depending on the configuration of the Ser364 side chain, the Ser364 hydroxyl group may function as a hydrogen-bond acceptor bonded to the Ser364 nitrogen at the 1 position (as shown in Fig. 3b), or as another hydrogen-bond donor to the ketone oxygen atom at the 2 position. A hydrogen bond may also form between Thr343 and the KAE609 fluorine atom at the 6′ position (Fig. 3b). H-F hydrogen bonds have been known to contribute to small-molecule binding in some contexts^23^, though some argue that they are rare and typically weak^24^.

### KAE609 potently blocks *Sc*Pma1p ATPase activity in a cell-free assay

The homology model suggested that KAE609 is a direct *Sc*Pma1p inhibitor. To test for direct inhibition, *Sc*Pma1p-coated vesicles were harvested from *S. cerevisiae* cells with a temperature-dependent defect in secretory-vesicle/plasma-membrane fusion and bearing a *ScPMA1* overexpression plasmid. This combination results in high levels of *Sc*Pma1p accumulation in vesicles with little detectable ATPase activity in empty vector controls or from mitochondrial or vacuolar ATPases. This ATPase assay measures the hydrolysis of ATP to Pi^25^. In these experiments, KAE609 had an IC_50_ of 81.1 nM (SE = 1.2 nM), ten times more potent than inorganic orthovanadate, the non-specific ATPase inhibitor used as a positive control (IC_50_ = 842 nM, SE = 1.20 nM, Fig. 3d). Artemisinin, an unrelated antimalarial that served as a negative control, showed no activity. KAE609 inhibition of *Sc*Pma1p in a cell-free context is consistent with the hypothesis that *Sc*Pma1p is the direct KAE609 target and inconsistent with an indirect mechanism by which *ScPMA1* mutations confer KAE609 resistance or an indirect mechanism by which KAE609 affects *Sc*Pma1p functioning.

**Figure 4:**
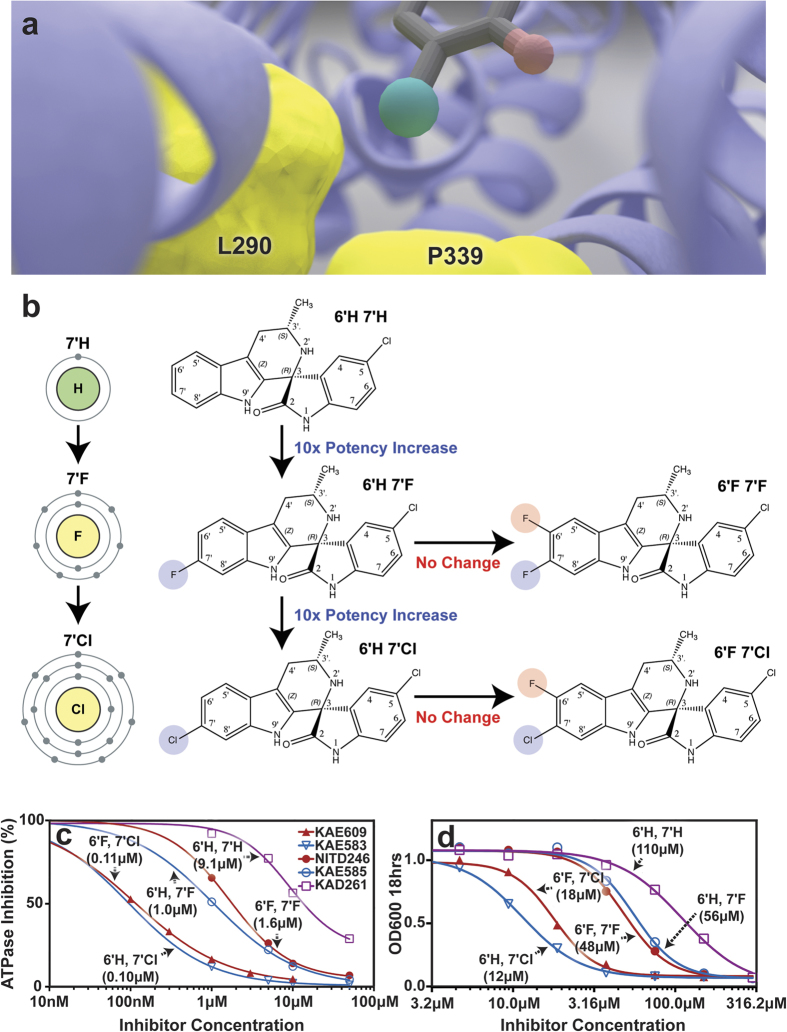
KAE609 halogenation. (**a**) An illustration of the predicted binding pose, which positions the 7′ chlorine atom (green) between two residues that were altered during directed evolution (L290S and P339T). Unlike the polar mutant residues, the nonpolar wild-type residues may stabilize the interaction with the 7′ chlorine atom. (**b**) In yeast, halogenation at the 7′ position (blue) has a substantial impact on potency, but halogenation at the 6′ position (red) has little impact. Note that KAE609 is the molecule designated 6′F 7′Cl. (**c**) The blue, red, and purple curves indicate 7′, 6′/7′, and no halogenation. In the cell-free assay, 7′ chlorination yields ~10- and ~100-fold greater potency than fluorination and no halogenation, respectively. (**d**) Structure-activity relationships showing correlation between direct inhibition of *Sc*PMA1p in the vesicle-based assay^25^ and activity in a whole-cell yeast replication assay (see methods). The *in vitro* IC_50_ values against blood-stage *P. falciparum* NF54 (in nM) were 13 ± 2.2 (6′H 7′H), 4.3 ± 0.4 (6′H 7′F), 0.33 ± 0.06 (6′F 7′F), 5.6 ± 0.7 (6′H 7′Cl), and 1.2 ± 0.2 (6′Cl 7′F, KAE609^72^. The following naming conventions were used by *Yeung et al*. in their paper concerning spiroindolone SAR: 6′H 7′H = (+)-1, 6′H 7′F = (+)-5, 6′F 7′F = (+)-6, 6′H 7′Cl = (+)-3, and 6′Cl 7′F (KAE609) = (+)-7^26^.

To verify the hypothesis that a hydrophobic interaction at the 7′ position is key to potency, we tested the activity of KAE609 derivatives^26^ with substitutions at both the 6′ and 7′ positions (Fig. 4a,b). In the cell-free *Sc*Pma1p assay (Fig. 4c), substituting fluorine for the chlorine at position 7′ results in a 10-fold decrease in potency. The removal of both the 6′ and 7′ halides causes a 100-fold decrease in potency. As the presence of fluorine at the 6′ position has little effect on potency, the removal of the 7′ halide appears to be exclusively responsible for the 100-fold decrease. Similar structure-activity relationships (SAR) are also observed in the whole-cell assay (Fig. 4d). The high degree of correlation between these two assays (r = 0.93) further supports our hypothesis that the cytotoxic effect of KAE609 is primarily due to direct *Sc*Pma1p inhibition.

## Discussion

Phenotypic screening is a powerful tool for discovering antiinfective and antiproliferative compounds^27^. Phenotypic cellular screens are efficient because they effectively multiplex many critical targets, identifying cytotoxic inhibitors of DNA replication, protein synthesis, secretion, transcription, or other cellular processes via a single, miniaturized assay. Although not necessarily critical for compound development, discovering a specific protein target can inform subsequent drug development, enabling both structure-guided drug design and a better understanding of off-target activity.

Existing techniques for target deconvolution are limited. For example, “omic methods” such as haploinsufficiency screens may produce an overwhelming amount of difficult-to-interpret data (e.g. at least 2,452 gene deletions confer resistance or sensitivity to cycloheximide, according to the *Saccharomyces* Genome Database)^28^. Proteomics approaches, which detect compound-binding proteins from a cell lysate, also produce high false-positive rates^29^, as do computational approaches such as proteome-wide virtual screening^30^. Additionally, transcriptional profiling is only rarely used for target identification^31^,^32^.

Directed evolution, on the other hand, has been used for many years to discover targets in bacteria. For example, the target of nalidixic acid was discovered to be DNA gyrase by mapping mutations in resistant lines^33^. More recently, directed evolution has also been used to find the targets of a novel antifungal^34^. However, its use has mostly relied on the availability of rapid methods for mapping the resistance locus to a given chromosomal position. The advent of whole-genome tiling arrays and whole-genome sequencing has made the method more practical in other single-celled organisms such as *Mycobacterium tuberculosis* bacilli^35^, malaria parasites^36^, trypanosomes^37^, and yeast^38^,^39^,^40^. Unlike other essential gene products that are often described as possible drug targets, drug targets discovered through directed evolution are known from the outset to be druggable and are considered “chemically validated.” The method has been successfully used to identify the targets of several antimalarial compounds initially found through phenotypic screens, as well as to identify genes contributing to the resistome^2^,^41^,^42^.

On the other hand, directed evolution followed by genome sequencing is still not practical in organisms that are multicellular, sexual, or difficult to culture. Many organisms also have lengthy cell cycles, large genomes that are prohibitively expensive to sequence, and/or multidrug efflux pumps that render them insensitive to many compounds of interest. Furthermore, resistance in multiploid organisms often requires simultaneous mutations in all alleles. Additionally, directed evolution is not possible for compounds that target non/low-replicative stages of pathogen life-cycles, such as next generation antimalarials currently under investigation that specifically target the liver or sexual stages. These compounds may, however, be cytotoxic to drug-sensitive yeast.

Here we demonstrate that directed evolution in surrogate species can identify the target class of a given drug, if not the actual target, opening up the method for use in other species. *S. cerevisiae* has advantages over many other organisms, including a well-characterized genome, a rich scientific literature, and a fast growth rate that produces resistant, engineered, or recombinant yeast in 24 hours versus 24 weeks (i.e., a nonconservative estimate of the time needed to perform selections and confirmation in pathogens such as *P. falciparum*). However, it should be acknowledged that yeast, even with a number of drug pumps removed, is still better at detoxifying xenobiotics than many microbes and that higher concentrations of compound will likely be needed to achieve inhibition.

Our data support the hypothesis that *Pf*Atp4p is the direct target of the spiroindolones and by extension the dihydroisoquinolones, which share the same binding site. We show direct inhibition of the *Sc*Pma1p enzyme in a cell-free system and study the SAR of the spiroindolones against their specific target, rather than the whole cell. We show that *Sc*Pma1p inhibition leads to a cytosolic decrease in pH, consistent with the hypothesis that *Sc*Pma1p is the KAE609 target. Our results are in agreement with studies showing that spiroindolones significantly reduce Na^+^-dependent ATPase activity in isolated membranes derived from parasitized erythrocytes^5^.

Our findings may also help explain the recent controversial observation that *PfATP4* mutants have either increased resistance or sensitivity to a broad range of unrelated antimalarials (reviewed in ref. 38), raising the possibility that *PfATP4* is a general drug resistance gene. The finding that a single amino acid change in *Sc*Pma1p sensitizes the cell to edelfosine by 750% suggests that these mutations confer a fitness cost to the protein, reducing either the ability to pump hydrogen ions or to stay associated with plasma-membrane lipid rafts. It stands to reason that amino-acid changes in *Pf*Atp4p may similarly affect function, leading to changes in cytosolic pH and membrane potential, with differing effects on xenobiotic uptake, deactivation, modification, degradation, or export.

An open question is whether or not *Pf*Atp4p and *Sc*Pma1p have the same function. The function of *Pf*Atp4p in malaria parasites remains uncertain, but experiments have shown that it may affect both sodium and proton homeostasis^5^. In contrast, *Sc*Pma1p is only involved in maintaining proton homeostasis. Although highly homologous (P(n) = 4.60E-38 per BLAST-P), *Pf*Atp4p and *Sc*Pma1p share only the E1-E2 ATPase domains, not the cation ATPase C terminal region (pfam00689) or the hydrolase domain (Fig. 1b). *Pf*Atp4p shows higher homology to *Sc*Pmr1p, *Sc*Ena1p, *Sc*Ena2p, and *Sc*Ena5p than to *Sc*Pma1p. However, with the exception of *Sc*Ena5p, these proteins are nonessential in *Saccharomyces* and thus are unlikely KAE609 targets. In addition, we show that KAE609 treatment results in acidification of the yeast cytosol, but alkalinization in *P. falciparum* was reported previously^5^. An explanation may be that both proteins pump hydrogen ions, but are in opposite orientation in the plasma membrane. This seems unlikely: although the orientation of *Pf*Atp4p in the plasma membrane is not known, it is likely that the ATP-binding domain, as with *Sc*Pma1p, is located in the cytosol^43^. Given that parasites lose the ability to extrude sodium after KAE609 treatment^5^, it seems likely that the two proteins do not have the same cellular function.

On the other hand, the *Pf*Atp4p binding pose is likely similar to the *Sc*Pma1p pose presented here, especially given that the evolved yeast mutations are in a highly conserved region of the protein that is shared with *P. falciparum*. Both the 6′ and 7′ halides were originally added during the drug optimization process to reduce CYP2C9-mediated hepatic clearance, and the discovery that these modifications also increase potency up to 45-fold in whole-cell malarial assays was fortuitous^26^. The current work suggests a structure-based explanation for the enhanced antimalarial activity of the halogenated compounds. However, further study of the malarial protein is warranted. There are some differences between the two proteins, as reflected in subtle differences in the ABC_16_-Monster and malarial whole-cell SAR. For example, 6′ halogenation has little effect on ABC_16_-Monster inhibition. In contrast, it increases potency against whole-cell *P. falciparum*^26^. Additionally, 7′ chlorination reduces malarial potency versus 7′ fluorination, but the opposite is true in yeast^26^.

Although our data strongly suggest that *Pf*Atp4p is the direct target of both the spiroindolones and, by analogy, the dihydroisoquinolones, it is not yet clear that *Pf*Atp4p is the direct target of the aminopyrazoles^6^ or pyrazole amides^9^. An aminopyrazole (GNF-Pf4492) was tested and had little measurable effect on ABC_16_-Monster growth (see Supplemental Table 3). Aminopyrazole resistance-conferring and -sensitizing mutations in *PfATP4* are distant from the sites identified in yeast. It is possible that these compounds bind to another pocket, or that these *PfATP4* mutations confer resistance through another mechanism.

Despite these questions, this work supports the hypothesis that *Pf*Atp4p is the direct KAE609 target in malaria and provides an additional method for characterizing P-type ATPase inhibitors. The data also support previous work suggesting that *Sc*Pma1p may be an attractive target for novel antifungals^44^,^45^,^46^. Recent successes in targeting P-type ATPases in *Plasmodium* may be translated into new strategies against other pathogens for which novel drug classes are badly needed.

## Methods

### Whole**-**Cell *S. cerevisiae* IC_50_ Assay

To measure compound activity against whole-cell yeast, each yeast generation was established using cells taken from single colonies on agar plates and inoculated into 2 mL of media in 5 mL snap-cap culture tubes. The tubes were cultured overnight at 250 RPM in a shaking incubator at 30 °C (Controlled Environment Incubator Shaker, Model G-25, New Brunswick Scientific Co., Inc.). Cultures were extracted when they were in mid-log, as judged by an OD600 (600 nm) reading between 0.1 and 0.5.

After being diluted to OD600 0.01, 30 μL of the cells were added to the wells of a 384 well plate. Fifteen 1:2 serial dilutions were subsequently performed, in duplicate for each clone, with starting IC_50_ values of 150 μM. Following an initial reading of OD600 (time 0 hours), the plate was placed in an incubator at 30 °C for 18 hours. After incubation, the plates were placed in a Synergy HT spectrophotometer, shaken for one minute on the “high” setting, and immediately read at OD600.

IC_50_ values were determined first by subtracting OD600 values at time 0 hours from time 18 hours. Nonlinear regression on log(inhibitor) vs. response with variable slope (four parameters) was then performed using Graphpad Prism. Minimum values were constrained to 0.0. For resistance assays, three biological replicates of technical duplicates were used to calculate each final IC_50_ value. P-values for IC_50_ fold-changes were determined using a one-tailed ratio paired t-test, comparing the ratio of the mutant and parental IC_50_ values. For the cross-resistance/cross-sensitivity assay, at least three biological replicates of technical triplicates were used to calculate each final IC_50_ value. P-values for IC_50_ fold-changes were determined using a two-tailed ratio paired t-test, since we did not have a prior hypothesis as to whether the IC_50_ should increase or decrease.

### Directed-Evolution Selection Protocol

ABC_16_-Monster cells (10 μL) were taken from a saturated culture and inoculated into 30 mL of YPD media in a 50 mL conical tube, with the desired KAE609 concentration. The tubes were then cultured at 250 RPM in a shaking incubator at 30 °C. Cultures that achieved OD600 values greater than 0.7 were advanced to the next generation of directed evolution.

### DNA Extraction, PCR, and Sanger Sequencing

DNA extractions were performed using the YeaStar Genomic DNA kit. To confirm all non-synonymous SNVs identified in open-reading frames by whole-genome sequencing, genomic DNA was PCR amplified in a 20 μL reaction volume with PrimeSTAR Max polymerase (Takara Bio Inc, Otsu, Shign, Japan) using the forward primer (5′-CTT CCA CTG TTA AGA GAG GTG AAG G-3′) and the reverse primer (5′-CTG GAA GCA GCC AAA CAA GCA GTC-3′) (Allele Biotechnology & Pharmaceuticals, Inc). PCR products were sequenced directly (Eton Bioscience Inc, San Diego, CA).

### Whole-Genome Sequencing and Analysis

Genomic DNA libraries were normalized to 0.2 ng/μL and prepared for sequencing using the Illumina Nextera XT kit whole-genome resequencing library, according to the manufacturer’s instructions (see the Illumina protocol of tagmentation followed by ligation, v. 2013, Illumina, Inc., San Diego). DNA libraries were clustered and run as 2 × 100 paired end reads on an Illumina HiSeq, according to the manufacturer’s instructions. Base calls were made using CASAVA v1.8.2. After sequencing, initial alignment was done using the Platypus software^47^. Briefly, reads were aligned to the reference *S. cerevisiae* genome using BWA, and unmapped reads were filtered using SAMTools. SNPs were then initially called using GATK and filtered using Platypus. Copy number variants were analyzed using Control-FreeC^48^. Sequencing statistics can be found in Supplementary Table 1.

### Site-Directed Mutagenesis

Point mutations were introduced in the ABC_16_-Monster strain using the Clustered Regularly Interspaced Short Palindromic Repeats (CRISPR) and CRISPR-associated (Cas) system from *Streptococcus pyogenes*^49^. Plasmids p414-TEF1p-Cas9-CYC1t (p414) and p426-SNR52p-gRNA.CAN1.Y-SUP4t (p426) were obtained from Addgene (Cambridge, MA). Plasmid transformation markers were replaced with markers that are compatible with the yeast strains used in the current study. The *TRP1* marker of the p414 plasmid was replaced with the *MET15* marker. As the centromere-autonomous replication sequence (CEN-ARS) region of this plasmid was difficult to amplify using Q5 DNA polymerase (New England Biolabs, Ipswich, MA), the CEN-ARS region of the original plasmid was also replaced with a CEN-ARS sequence from *Mycoplasma mycoides* JCVI-syn1.0^50^. To create a p414 fragment containing the mycoplasma CEN-ARS sequence, three PCR products were generated: 1) an upstream 289-base-pair fragment amplified from p414 using the primers 414_VecF and 414_GB_3 R, 2) a 525-base-pair CEN-ARS fragment amplified from genomic DNA of the mycoplasma strain using the primers 414_GB_2 F and 414_GB_2 R, and 3) a downstream 644-base-pair fragment amplified from p414 using the primers 414_GB_1 F and Pre-p426_Halfway_R (see Supplementary Table 4). These three fragments were combined using crossover PCR to generate a single, contiguous fragment (fragment 1). To prepare the remaining parts, a 2.1-kb fragment containing the *MET15* gene was amplified from a *MET15*-containing plasmid^51^ using the primers 414_MET15F and New_414_MET15_R (fragment 2). The two additional fragments generated from the p414 plasmid were a 1.7-kb fragment amplified using the primers p426_Halfway_F and New_414_Vec_Only_HalfR (fragment 3), and a 5.6-kb fragment amplified using the primers 414_Vec_Half_F and 414_VecR (fragment 4). Fragments 1–4 were combined using homologous recombination in yeast to make the plasmid p414-TEF1p-Cas9-CYC1t-MET15.

The *URA3* marker of the p426 guide RNA plasmid was replaced with the *LEU2* marker. The p426 backbone was amplified as two fragments, a 2.2-kb fragment amplified with the primers Attempt2_426_Vec_F and p426_Halfway_R (fragment 5), and a 3.1-kb fragment amplified with the primers p426_Halfway_F and New_426_Vec_R (fragment 6). A 2.1-kb *LEU2* fragment was amplified from a plasmid derived from pRS315 (ref. 52; AM & YS, unpublished result) using the primers New_426_LEU2_F and Attempt2_426_LEU2_F (fragment 7). Fragments 5–7 were combined using Gibson assembly^53^ to make the plasmid p426-SNR52p-gRNA.CAN.Y-SUP4t-LEU2.

For the introduction of each mutation, a unique 20-base-pair guide RNA (gRNA) sequence was required. To generate the required variants of the gRNA plasmid, p426-SNR52p-gRNA.CAN.Y-SUP4t-LEU2 was PCR-amplified using a high-fidelity DNA polymerase PrimeSTAR Max (2 × Master Mix, Takara Bio, Mountain View, CA) and primers containing the appropriate 20-base-pair gRNA sequence at the 5′ end (see Supplementary Table 5). The two ends of the PCR product shared 25 base pairs of homology, replacing the original gRNA sequence.

After purification using NucleoSpin Gel and the PCR Clean-up kit (Macherey-Nagel, Bethlehem, PA), the linearized plasmid was introduced into High Efficiency NEB 5-alpha chemically competent cells (New England Biolabs, Ipswich, MA), per the manufacturer’s protocol^54^,^55^. Transformed colonies were selected on LB-ampicillin agar plates. Colonies were then cultured in liquid LB-ampicillin medium, and plasmids were isolated using a miniprep kit (Qiagen, Valencia, CA). The correct gRNA sequence was verified using Sanger sequencing. Repair fragments were prepared from complementary 90-base-pair oligonucleotides (IDT, Coralville, IA), which included the desired mutation (see Supplementary Table 6). To prepare double-stranded repair fragments, complementary oligonucleotides were mixed in equimolar amounts, denatured at 100 °C for five minutes, and allowed to cool to 25 °C with a ramp rate of 0.1 °C per second.

The ABC_16_-Monster strain was initially transformed with ~200 ng of the p414-TEF1p-Cas9-CYC1t-MET15 plasmid using a standard lithium acetate transformation method^56^ and selected on synthetic complete (SC) minus Met agar plates. To introduce a desired mutation, Cas9-expressing cells were cultured overnight in SC-Met. Using lithium acetate transformation^56^, the cells were co-transformed with ~500 ng of the appropriate gRNA plasmid and 2.5 nmol of the corresponding double-stranded repair fragment. All of the transformed cells were selected on SC-Met-Leu agar plates.

To verify the correct introduction of the mutations, the genomic region around the mutation was PCR-amplified using a high-fidelity DNA polymerase Q5 (2 × Master Mix, New England Biolabs) and sequenced using Sanger sequencing (see Supplementary Table 7). Before phenotype testing, strains were cultured overnight in a YPDA liquid medium, the resulting cells were plated on a YPDA agar medium to form isolated colonies, and the loss of both plasmids was confirmed by transferring the colonies to both rich and selective media.

### *Sc*Pma1p ATPase Assay

ATP hydrolysis was assayed at 30 °C in 0.5 mL of 50 mM MES/Tris, pH 6.25, 5 mM NaN_3_, 5 mM Na_2_ ATP (Roche), 10 mM MgCl_2_, and an ATP regenerating system (5 mM phosphoenolpyruvate and 50 μg/mL pyruvate kinase). The reaction was terminated after 20 minutes by the addition of Fiske and Subbarow reagent, and the release of inorganic phosphate was measured at 660 nm after 45 minutes of color development^25^.

### Cytosolic pH Measurement

To measure cytosolic pH-changes the yeast strains containing a cytoplasmically expressed ratiometric pHluorin plasmid (UCC9633) was used (see Supplemental Table 8). The plasmid was created by amplifying a fragment of plasmid pADH1pr-RMP^57^ using primers URA3-tTA-intChr1 F and R to integrate ratiometric pHluorin^19^ into an empty region of chromosome 1 (17068–17161) under the control of the ADH1 promoter. Cells were cultured in YEPD (1% yeast extract, 2% peptone, 2% glucose) exponentially for 4 hours prior to 3 hours of treatment with 200 uM KAE609. For pH measurement, cells were transferred to low fluorescence medium^58^ at a density of 3 × 10^7 ^cells/mL after rinsing them in an equal volume of low fluorescence medium. Calibration curves were calculated as described in ref. 57.

To quantify cytosolic pH and generate calibration curves, pHluorin fluorescence emission was measured at 512 nm using a SpectraMax M5 microtitre plate spectrofluorometer (Molecular Devices, Sunnyvale, CA), providing excitation bands of 9 nm centered around 390 and 470 nm. Background fluorescence for wild-type cells not expressing pHluorin (UCC4925) was subtracted from the measurements. The ratio of emission intensity resulting from excitation at 390 and 470 nm was calculated. Ratios were fitted to a calibration curve to derive cytosolic pH from three biological replicates for each pH measurement. pH values are represented as mean ± sd.

### *Sc*Pma1p Homology Modeling

To better understand the KAE609 binding pose, a homology model of *Sc*Pma1p was built with Schrödinger’s Prime software^59^ using the UniProt^60^ sequence P05030 and a structure of the highly homologous *Sus scrofa* sodium-potassium pump (PDBID: 3N2F, chain C)^61^. The 3N2F and P05030 amino-acid sequences were first aligned using ClustalW^62^. The model was then constructed using Schrödinger’s knowledge-based method. The structure was further processed with Schrödinger’s Protein Preparation Wizard^63^. Hydrogen atoms were added at pH 7.0 using PROPKA^64^, water molecules were removed, and disulfide bonds were appropriately modeled. The system was then subjected to a restrained minimization using the OPLS_2005 forcefield^65^,^66^, converging the heavy atoms to an RMSD of 0.30 Å.

### KAE609 Docking

A three-dimensional KAE609 model was prepared using Schrodinger’s LigPrep module. The protonation state was determined for pH values in the 5.0–9.0 range using Epik^67^. The most likely tautomeric state and low-energy ring conformations were similarly determined. The molecular geometry was optimized using the OPLS_2005 forcefield^65^,^66^.

Glide XP^20^,^21^ was used to dock KAE609 into the modeled E2-*Sc*Pma1p pocket near the amino acids associated with evolved resistance. The ligand diameter midpoint box was 14 Å cubed. Glide was instructed to use flexible ligand sampling, to sample nitrogen inversions and ring conformations, to bias torsion sampling for amides only, and to add Epik state penalties to the docking scores. The van der Waals radii of atoms with assigned partial charges less than or equal to 0.15 e were scaled to 80%.

## Additional Information

**Accession codes:** Sequences have been placed in the short read sequence archive (http://www.ncbi.nlm.nih.gov/sra) under Accession code SRP074482.

**How to cite this article**: Goldgof, G. M. *et al*. Comparative chemical genomics reveal that the spiroindolone antimalarial KAE609 (Cipargamin) is a P-type ATPase inhibitor. *Sci. Rep.* **6**, 27806; doi: 10.1038/srep27806 (2016).

## Supplementary Material

Supplementary Information

## Figures and Tables

**Table 1:** Nonsynonymous changes identified by whole-genome sequencing.

**Selection**	**Gene Name**	**Nucl. change**	**AA Change**	**Description**
Lineage 1	*PMA1*	Cca/Aca	P339T	Plasma membrane P2-type H^+^ -ATPase
Lineage 1	*YOS9*	aaC/aaA	N490K	ER quality-control lectin
Lineage 1	*YRR1*	ttG/ttC	L611F	Zn2-Cys6 zinc-finger transcription factor
Lineage 2	*PMA1*	aaC/aaG	N290S	Plasma membrane P2-type H^+^-ATPase
Lineage 2	*SLA1*	Aat/Gat	N788D	Cytoskeletal protein binding protein
Lineage 2	*YRR1*	aCg/aAg	T623K	Zn2-Cys6 zinc-finger transcription factor
Lineage 3	*GCD1*	Gtt/Ctt	V450L	Gamma subunit of the translation init. factor eIF2B;
Lineage 3	*PMA1*	Ggt/Agt	G294S	Plasma membrane P2-type H^+^-ATPase

Clones were sequenced to 40–90X coverage using paired-end reads and aligned to the S288c reference genome. *PMA1* mutations are shown in bold. No intergenic mutations near *PMA1* were identified. In addition, PCR analysis of nonclonal cultures identified an additional L291K *PMA1* substitution in Lineage 2, Round 5, derived from a parent containing the *YRR1* L611F mutation. This genotype was confirmed by whole-genome sequencing. Nonsynonymous coding changes in retrotransposons and flocculation genes (*FLO1, FLO4, FLO2, FLO9*) were also observed but were considered nonspecific.

## References

[b1] White N. J. . Spiroindolone KAE609 for Falciparum and Vivax Malaria. New Engl. J. Med . 371, 403–410, doi: 10.1056/NEJMoa1315860 (2014). 25075833 PMC4143746

[b2] Rottmann M. . Spiroindolones, a Potent Compound Class for the Treatment of Malaria. Science 329, 1175–1180, doi: 10.1126/Science.1193225 (2010). 20813948 PMC3050001

[b3] Leong F. J. . A First-in-Human Randomized, Double-Blind, Placebo-Controlled, Single- and Multiple-Ascending Oral Dose Study of Novel Antimalarial Spiroindolone KAE609 (Cipargamin) To Assess Its Safety, Tolerability, and Pharmacokinetics in Healthy Adult Volunteers. Antimicrob Agents Ch 58, 6209–6214, doi: 10.1128/Aac.03393-14 (2014). PMC4187895 25114127

[b4] van Pelt-Koops J. C. . The Spiroindolone Drug Candidate NITD609 Potently Inhibits Gametocytogenesis and Blocks Plasmodium falciparum Transmission to Anopheles Mosquito Vector. Antimicrob Agents Ch 56, 3544–3548, doi: 10.1128/Aac.06377-11 (2012). PMC3393464 22508309

[b5] Spillman N. J. . Na+ Regulation in the Malaria Parasite Plasmodium falciparum Involves the Cation ATPase PfATP4 and Is a Target of the Spiroindolone Antimalarials. Cell Host Microbe 13, 227–237, doi: 10.1016/j.chom.2012.12.006 (2013). 23414762 PMC3574224

[b6] Flannery E. L. . Mutations in the P-Type Cation-Transporter ATPase 4, PfATP4, Mediate Resistance to Both Aminopyrazole and Spiroindolone Antimalarials. ACS Chem. Biol. 10, 413–420, doi: 10.1021/cb500616x (2015). 25322084 PMC4340351

[b7] Jimenez-Diaz M. B. . (+)-SJ733, a clinical candidate for malaria that acts through ATP4 to induce rapid host-mediated clearance of Plasmodium. Proc. Natl. Acad. Sci. USA. 111, E5455–E5462, doi: 10.1073/pnas.1414221111 (2014). 25453091 PMC4273362

[b8] Lehane A. M., Ridgway M. C., Baker E. & Kirk K. Diverse chemotypes disrupt ion homeostasis in the malaria parasite. Mol. Microbiol. 94, 327–339, doi: 10.1111/mmi.12765 (2014). 25145582

[b9] Vaidya A. B. . Pyrazoleamide compounds are potent antimalarials that target Na+ homeostasis in intraerythrocytic Plasmodium falciparum. Nat. Commun. 5, doi: ARTN 5521 10.1038/ncomms6521 (2014). PMC4263321 25422853

[b10] Suzuki Y. . Knocking out multigene redundancies via cycles of sexual assortment and fluorescence selection. Nat. Methods 8, 159–164, doi: 10.1038/nmeth.1550 (2011). 21217751 PMC3076670

[b11] Serrano R., Kiellandbrandt M. C. & Fink G. R. Yeast Plasma-Membrane Atpase Is Essential for Growth and Has Homology with (Na++K+), K+- and Ca-2+ -Atpases. Nature 319, 689–693, doi: 10.1038/319689a0 (1986). 3005867

[b12] Cuesta-Marban A. . Drug uptake, lipid rafts, and vesicle trafficking modulate resistance to an anticancer lysophosphatidylcholine analogue in yeast. J Biol Chem 288, 8405–8418, doi: 10.1074/jbc.M112.425769 (2013). 23335509 PMC3605657

[b13] Czyz O. . Alteration of plasma membrane organization by an anticancer lysophosphatidylcholine analogue induces intracellular acidification and internalization of plasma membrane transporters in yeast. J Biol Chem 288, 8419–8432, doi: 10.1074/jbc.M112.425744 (2013). 23344949 PMC3605658

[b14] Mahadeo M. . Disruption of lipid domain organization in monolayers of complex yeast lipid extracts induced by the lysophosphatidylcholine analogue edelfosine *in vivo*. Chemistry and physics of lipids 191, 153–162, doi: 10.1016/j.chemphyslip.2015.09.004 (2015). 26386399

[b15] Zaremberg V., Gajate C., Cacharro L. M., Mollinedo F. & McMaster C. R. Cytotoxicity of an anti-cancer lysophospholipid through selective modification of lipid raft composition. J Biol Chem 280, 38047–38058, doi: 10.1074/jbc.M502849200 (2005). 16155007

[b16] Cyert M. S. & Philpott C. C. Regulation of cation balance in Saccharomyces cerevisiae. Genetics 193, 677–713, doi: 10.1534/genetics.112.147207 (2013). 23463800 PMC3583992

[b17] McCusker J. H., Perlin D. S. & Haber J. E. Pleiotropic plasma membrane ATPase mutations of Saccharomyces cerevisiae. Mol. Cell Biol. 7, 4082–4088 (1987). 2963211 10.1128/mcb.7.11.4082-4088.1987 PMC368079

[b18] Portillo F. & Serrano R. Growth control strength and active site of yeast plasma membrane ATPase studied by site-directed mutagenesis. European journal of biochemistry / FEBS 186, 501–507 (1989). 10.1111/j.1432-1033.1989.tb15235.x 2532597

[b19] Miesenbock G., De Angelis D. A. & Rothman J. E. Visualizing secretion and synaptic transmission with pH-sensitive green fluorescent proteins. Nature 394, 192–195, doi: 10.1038/28190 (1998). 9671304

[b20] Repasky M. P., Shelley M. & Friesner R. A. Flexible ligand docking with Glide. Curr. Protoc. Bioinformatics Chapter 8, Unit 8 12, doi: 10.1002/0471250953.bi0812s18 (2007). 18428795

[b21] Friesner R. A. . Extra precision glide: Docking and scoring incorporating a model of hydrophobic enclosure for protein-ligand complexes. J. Med. Chem. 49, 6177–6196, doi: 10.1021/Jm051256o (2006). 17034125

[b22] Halgren T. A. . Glide: a new approach for rapid, accurate docking and scoring. 2. Enrichment factors in database screening. J. Med. Chem. 47, 1750–1759 (2004). 15027866 10.1021/jm030644s

[b23] Hao G. F. . Computational Discovery of Picomolar Q(o) Site Inhibitors of Cytochrome bc(1) Complex. J. Am. Chem. Soc. 134, 11168–11176, doi: 10.1021/Ja3001908 (2012). 22690928

[b24] Dunitz J. D. & Taylor R. Organic fluorine hardly ever accepts hydrogen bonds. Chem. - Eur. J . 3, 89–98, doi: 10.1002/Chem.19970030115 (1997).

[b25] Ambesi A., Allen K. E. & Slayman C. W. Isolation of transport-competent secretory vesicles from Saccharomyces cerevisiae. Anal. Biochem. 251, 127–129, doi: 10.1006/abio.1997.2257 (1997). 9300097

[b26] Yeung B. K. S. . Spirotetrahydro beta-Carbolines (Spiroindolones): A New Class of Potent and Orally Efficacious Compounds for the Treatment of Malaria. J. Med. Chem. 53, 5155–5164, doi: 10.1021/jm100410f (2010). 20568778 PMC6996867

[b27] Wagner B. K. & Schreiber S. L. The Power of Sophisticated Phenotypic Screening and Modern Mechanism-of-Action Methods. Cell Chemical Biology 10.1016/j.chembiol.2015.11.008 (2016). PMC4779180 26933731

[b28] Cherry J. M. . Saccharomyces Genome Database: the genomics resource of budding yeast. Nucleic Acids Res. 40, D700–D705, doi: 10.1093/nar/gkr1029 (2012). 22110037 PMC3245034

[b29] Burdine L. & Kodadek T. Target identification in chemical genetics: the (often) missing link. Chem. Biol. 11, 593–597, doi: 10.1016/j.chembiol.2004.05.001 (2004). 15157870

[b30] Durrant J. D. . A multidimensional strategy to detect polypharmacological targets in the absence of structural and sequence homology. PLoS Comput. Biol. 6, e1000648, doi: 10.1371/journal.pcbi.1000648 (2010). 20098496 PMC2799658

[b31] Palchaudhuri R. & Hergenrother P. J. Transcript profiling and RNA interference as tools to identify small molecule mechanisms and therapeutic potential. ACS Chem Biol 6, 21–33, doi: 10.1021/cb100310h (2011). 21105689 PMC3121534

[b32] Mohr S. E., Smith J. A., Shamu C. E., Neumuller R. A. & Perrimon N. RNAi screening comes of age: improved techniques and complementary approaches. Nat Rev Mol Cell Biol 15, 591–600, doi: 10.1038/nrm3860 (2014). 25145850 PMC4204798

[b33] Hane M. W. & Wood T. H. Escherichia coli K-12 mutants resistant to nalidixic acid: genetic mapping and dominance studies. Journal of Bacteriology 99, 238–241 (1969). 4895844 10.1128/jb.99.1.238-241.1969 PMC249993

[b34] Rock F. L. . An antifungal agent inhibits an aminoacyl-tRNA synthetase by trapping tRNA in the editing site. Science 316, 1759–1761, doi: 10.1126/science.1142189 (2007). 17588934

[b35] Andries K. . A diarylquinoline drug active on the ATP synthase of Mycobacterium tuberculosis. Science 307, 223–227, doi: 10.1126/science.1106753 (2005). 15591164

[b36] Baragana B. . A novel multiple-stage antimalarial agent that inhibits protein synthesis. Nature 522, 315–320, doi: 10.1038/nature14451 (2015). 26085270 PMC4700930

[b37] Khare S. . Utilizing Chemical Genomics to Identify Cytochrome b as a Novel Drug Target for Chagas Disease. PLoS Pathog 11, e1005058, doi: 10.1371/journal.ppat.1005058 (2015). 26186534 PMC4506092

[b38] Spillman N. J. & Kirk K. The malaria parasite cation ATPase PfATP4 and its role in the mechanism of action of a new arsenal of antimalarial drugs. Int. J. Parasitol. Drugs Drug Resist . 5, 149–162, doi: 10.1016/j.ijpddr.2015.07.001 (2015). 26401486 PMC4559606

[b39] Ojini I. & Gammie A. Rapid Identification of Chemoresistance Mechanisms Using Yeast DNA Mismatch Repair Mutants. G3 (Bethesda, Md.) 5, 1925–1935, doi: 10.1534/g3.115.020560 (2015). 26199284 PMC4555229

[b40] Wride D. A. . Confirmation of the cellular targets of benomyl and rapamycin using next-generation sequencing of resistant mutants in S. cerevisiae. Molecular bioSystems 10, 3179–3187, doi: 10.1039/c4mb00146j (2014). 25257345 PMC4653042

[b41] Hoepfner D. . Selective and Specific Inhibition of the Plasmodium falciparum Lysyl-tRNA Synthetase by the Fungal Secondary Metabolite Cladosporin. Cell Host Microbe 11, 654–663, doi: 10.1016/J.Chom.2012.04.015 (2012). 22704625 PMC3391680

[b42] McNamara C. W. . Targeting Plasmodium PI(4)K to eliminate malaria. Nature 504, 248-+, doi: 10.1038/Nature12782 (2013). 24284631 PMC3940870

[b43] Kuhlbrandt W. Biology, structure and mechanism of P-type ATPases. Nat Rev Mol Cell Biol 5, 282–295, doi: 10.1038/nrm1354 (2004). 15071553

[b44] Monk B. C. . Surface-active fungicidal D-peptide inhibitors of the plasma membrane proton pump that block azole resistance. Antimicrob. Agents Chemother. 49, 57–70, doi: 10.1128/AAC.49.1.57-70.2005 (2005). 15616276 PMC538910

[b45] Perlin D. S., Seto-Young D. & Monk B. C. The plasma membrane H(+)-ATPase of fungi. A candidate drug target? Ann N Y Acad Sci 834, 609–617 (1997). 9405872 10.1111/j.1749-6632.1997.tb52330.x

[b46] Seto-Young D., Monk B., Mason A. B. & Perlin D. S. Exploring an antifungal target in the plasma membrane H(+)-ATPase of fungi. Biochim. Biophys. Acta 1326, 249–256 (1997). 9218555 10.1016/s0005-2736(97)00028-x

[b47] Manary M. J. . Identification of pathogen genomic variants through an integrated pipeline. Bmc Bioinformatics 15, doi: Artn 63 10.1186/1471-2105-15-63 (2014). PMC3945619 24589256

[b48] Boeva V. . Control-FREEC: a tool for assessing copy number and allelic content using next-generation sequencing data. Bioinformatics 28, 423–425, doi: 10.1093/bioinformatics/btr670 (2012). 22155870 PMC3268243

[b49] DiCarlo J. E. . Genome engineering in Saccharomyces cerevisiae using CRISPR-Cas systems. Nucleic Acids Res. 41, 4336–4343, doi: 10.1093/nar/gkt135 (2013). 23460208 PMC3627607

[b50] Gibson D. G. . Creation of a bacterial cell controlled by a chemically synthesized genome. Science 329, 52–56, doi: 10.1126/science.1190719 (2010). 20488990

[b51] Suzuki Y. . Bacterial genome reduction using the progressive clustering of deletions via yeast sexual cycling. Genome Res. 25, 435–444, doi: 10.1101/gr.182477.114 (2015). 25654978 PMC4352883

[b52] Sikorski R. S. & Hieter P. A system of shuttle vectors and yeast host strains designed for efficient manipulation of DNA in Saccharomyces cerevisiae. Genetics 122, 19–27 (1989). 2659436 10.1093/genetics/122.1.19 PMC1203683

[b53] Gibson D. G. . Enzymatic assembly of DNA molecules up to several hundred kilobases. Nat. Methods 6, 343–345, doi: 10.1038/nmeth.1318 (2009). 19363495

[b54] Bugyi B. [Sir Joseph Barcroft and the Cambridge physiology school]. Orv. Hetil. 113, 2663–2665 (1972). 4562628

[b55] Kostylev M., Otwell A. E., Richardson R. E. & Suzuki Y. Cloning Should Be Simple: Escherichia coli DH5alpha-Mediated Assembly of Multiple DNA Fragments with Short End Homologies. PLoS One 10, e0137466, doi: 10.1371/journal.pone.0137466 (2015). 26348330 PMC4562628

[b56] Schiestl R. H. & Gietz R. D. High efficiency transformation of intact yeast cells using single stranded nucleic acids as a carrier. Curr. Genet. 16, 339–346 (1989). 2692852 10.1007/BF00340712

[b57] Henderson K. A., Hughes A. L. & Gottschling D. E. Mother-daughter asymmetry of pH underlies aging and rejuvenation in yeast. Elife 3, e03504, doi: 10.7554/eLife.03504 (2014). 25190112 PMC4175738

[b58] Orij R., Postmus J., Ter Beek A., Brul S. & Smits G. J. *In vivo* measurement of cytosolic and mitochondrial pH using a pH-sensitive GFP derivative in Saccharomyces cerevisiae reveals a relation between intracellular pH and growth. Microbiology 155, 268–278, doi: 10.1099/mic.0.022038-0 (2009). 19118367

[b59] Jacobson M. P. . A Hierarchical Approach to All-Atom Protein Loop Prediction. Proteins: Struct. Funct. Bioinf . 55, 351–367 (2004). 10.1002/prot.10613 15048827

[b60] Bairoch A. . The Universal Protein Resource (UniProt). Nucleic Acids Res. 33, D154–159, doi: 10.1093/nar/gki070 (2005). 15608167 PMC540024

[b61] Laursen M., Gregersen J. L., Yatime L., Nissen P. & Fedosova N. U. Structures and characterization of digoxin- and bufalin-bound Na+, K+-ATPase compared with the ouabain-bound complex. Proc. Natl. Acad. Sci. USA 112, 1755–1760, doi: 10.1073/Pnas.1422997112 (2015). 25624492 PMC4330780

[b62] Larkin M. A. . Clustal W and Clustal X version 2.0. Bioinformatics 23, 2947–2948, doi: btm404 [pii] 10.1093/bioinformatics/btm404 (2007). 17846036 10.1093/bioinformatics/btm404

[b63] Sastry G. M., Adzhigirey M., Day T., Annabhimoju R. & Sherman W. Protein and ligand preparation: parameters, protocols, and influence on virtual screening enrichments. J. Comput. Aid. Mol. Des . 27, 221–234, doi: 10.1007/S10822-013-9644-8 (2013). 23579614

[b64] Olsson M. H. M., Sondergaard C. R., Rostkowski M. & Jensen J. H. PROPKA3: Consistent Treatment of Internal and Surface Residues in Empirical pK(a) Predictions. J. Chem. Theory Comput. 7, 525–537, doi: 10.1021/Ct100578z (2011). 26596171

[b65] Jorgensen W. L., Maxwell D. S. & TiradoRives J. Development and testing of the OPLS all-atom force field on conformational energetics and properties of organic liquids. J. Am. Chem. Soc. 118, 11225–11236 (1996).

[b66] Kaminski G. A., Friesner R. A., Tirado-Rives J. & Jorgensen W. L. Evaluation and reparametrization of the OPLS-AA force field for proteins via comparison with accurate quantum chemical calculations on peptides. J. Phys. Chem. B 105, 6474–6487, doi: 10.1021/Jp003919d (2001).

[b67] Shelley J. C. . Epik: a software program for pK(a) prediction and protonation state generation for drug-like molecules. J. Comput. Aid. Mol. Des . 21, 681–691, doi: 10.1007/s10822-007-9133-z (2007). 17899391

[b68] Altschul S. F. . Gapped BLAST and PSI-BLAST: a new generation of protein database search programs. Nucleic Acids Res. 25, 3389–3402 (1997). 9254694 10.1093/nar/25.17.3389 PMC146917

[b69] Kuhlbrandt W., Zeelen J. & Dietrich J. Structure, mechanism, and regulation of the neurospora plasma membrane H+-ATPase. Science 297, 1692–1696, doi: 10.1126/Science.1072574 (2002). 12169656

[b70] Jo S., Lim J. B., Klauda J. B. & Im W. CHARMM-GUI Membrane Builder for mixed bilayers and its application to yeast membranes. Biophys. J. 97, 50–58, doi: S0006-3495(09)00791-7 [pii] 10.1016/j.bpj.2009.04.013 (2009). 19580743 PMC2711372

[b71] Lomize M. A., Lomize A. L., Pogozheva I. D. & Mosberg H. I. OPM: Orientations of proteins in membranes database. Bioinformatics 22, 623–625, doi: 10.1093/Bioinformatics/Btk023 (2006). 16397007

[b72] Lakshminarayana S. B. . Pharmacokinetic-pharmacodynamic analysis of spiroindolone analogs and KAE609 in a murine malaria model. Antimicrob. Agents Chemother. 59, 1200–1210, doi: 10.1128/aac.03274-14 (2015). 25487807 PMC4335872

