# Supplementary material for: Comparative chemical genomics reveal that the spiroindolone antimalarial KAE609 (Cipargamin) is a P-type ATPase inhibitor
Source: Sci Rep. 2016 Jun 13;6:27806. doi: 10.1038/srep27806 (PMC4904242; doi:10.1038/srep27806)
Supplement: Supplementary Information [file srep27806-s1.doc]

# Comparative chemical genomics reveal that the spiroindolone antimalarial KAE609 (Cipargamin) is a P-type ATPase inhibitor

Gregory M. Goldgof^1,2,8^, Jacob D. Durrant^3,8^, Sabine Ottilie^1^, Edgar Vigil^1^, Kenneth E. Allen^4^, Felicia Gunawan^1^, Maxim Kostylev^2^, Kiersten A. Henderson^5^, Jennifer Yang^1^, Jake Schenken^1^, Gregory M. LaMonte^1^, Micah J. Manary^1^, Ayako Murao^2^, Marie Nachon^1^, Rebecca Stanhope^1^, Maximo Prescott^1^, Case W. McNamara^6,7^, Carolyn W. Slayman^4^, Rommie E. Amaro^3^, Yo Suzuki^2^ & Elizabeth A. Winzeler^1*^

^1^ Division of Pharmacology and Drug Discovery, Department of Pediatrics, University of California, San Diego, School of Medicine, La Jolla, California, USA

^2^ Department of Synthetic Biology and Bioenergy, J. Craig Venter Institute, La Jolla, California, USA

^3^ Department of Chemistry & Biochemistry and the National Biomedical Computation Resource, University of California, San Diego, La Jolla, California, USA

^4^ Department of Genetics, Yale University School of Medicine, New Haven, Connecticut, USA.

^5^ Basic Sciences Division, Fred Hutchinson Cancer Research Center, Seattle, WA, USA

^6^ Genomics Institute of the Novartis Research Foundation, San Diego, California, USA

^7^ Current address: California Institute for Biomedical Research (Calibr), La Jolla, California, USA

^8^ These authors contributed equally to this work.

* e-mail: [ewinzeler@ucsd.edu](mailto:ewinzeler@ucsd.edu)

# Supporting Information

|  | **Lineage 1** | **Lineage 2** | **Lineage 3** |
| --- | --- | --- | --- |
| **Total reads** | 11054710 | 14960010 | 5091610 |
| **Aligned reads** | 10879244 | 14776306 | 5008606 |
| **Percent Aligned Reads** | 98.4127 | 98.772 | 98.3698 |
| **Mean Coverage** | 74.26 | 89.41 | 40.50 |
| **Percent bases covered by 5 or more reads** | 99.4 | 99.4 | 99.3 |
| **Mean read length** | 100 | 100 | 100 |
| **Median insert size** | 144 | 101 | 307 |
| **Total SNVs** | 8 | 5 | 5 |
| **Intergenic SNVs** | 3 | 2 | 3 |
| **Missense/non-synonymous** | 3 | 3 | 2 |
| **Mutated Genes** | PMA1, YRR1, YOS9 | PMA1, YRR1, SLA1 | PMA1, GCD1 |
| **Insertions Relative to Wild-Type*** | *URA3* (16x), *CYC1* (3-4x) | *URA3* (16x), *CYC1* (3-4x) | *URA3* (16x), *CYC1* (3-4x) |
| **Deletions Relative to Wild-Type**** | *adp1∆ snq2∆ ycf1∆ pdr15∆ yor1∆ vmr1∆ pdr11∆ nft1∆ bpt1∆ ybt1∆ ynr070w∆ yol075c∆ aus1∆ pdr5∆ pdr10∆ pdr12∆* | *adp1∆ snq2∆ ycf1∆ pdr15∆ yor1∆ vmr1∆ pdr11∆ nft1∆ bpt1∆ ybt1∆ ynr070w∆ yol075c∆ aus1∆ pdr5∆ pdr10∆ pdr12∆* | *adp1∆ snq2∆ ycf1∆ pdr15∆ yor1∆ vmr1∆ pdr11∆ nft1∆ bpt1∆ ybt1∆ ynr070w∆ yol075c∆ aus1∆ pdr5∆ pdr10∆ pdr12∆* |
| **Insertions Relative to ABC_16_-Monster (Parent)** | none | none | none |
| **Deletions Relative to ABC_16_-Monster (Parent)** | none | none | none |

**Supplementary Table 1: Whole-genome sequencing statistics for KAE609 resistant mutants.** Sample taxonomic designation: *Saccharomyces cerevisiae.* Platform used: Illumina_HiSeq Paired End Reads. Reference used (md5): S288C_R64-1-1 (3571059465). All were sequenced after round 5 of selection.

*Our pipeline did not include the analysis of insertions that aligned to other species, but selection markers from other species should also theoretically be present. The ABC_16_-Monster and all of its derived strains have a defective version of the HO gene, whereas the wild-type strain has a full deletion. This difference is not included in the table, for the sake of clarity. **The 16 deletions in all strains are the 16 ABC-transporters expected to be absent.

Note: Mitochondrial DNA was not analyzed due to poor coverage.

| **Compound/Drug** | **Resistant Clones** | **# Nonsyn. SNVs in PMA1** | **# Nonsyn. SNVs in YRR1** |
| --- | --- | --- | --- |
| Artemisinin | 4 | 0 | 0 |
| CHEMBL524973 | 4 | 0 | 0 |
| CHEMBL528165 | 4 | 0 | 0 |
| CHEMBL531401 | 3 | 0 | 0 |
| CHEMBL547770 | 3 | 0 | 0 |
| CHEMBL578293 | 2 | 0 | 0 |
| CHEMBL585357 | 2 | 0 | 1 |
| CHEMBL586184 | 5 | 0 | 2 |
| CHEMBL586701 | 3 | 0 | 0 |
| CHEMBL598478 | 5 | 0 | 0 |
| CHEMBL601580 | 2 | 0 | 0 |
| CHEMBL606167 | 3 | 0 | 0 |
| Cycloheximide | 3 | 0 | 0 |
| GNF179^1^ | 10 | 0 | 0 |
| KAE609 | 3 | 4 | 2 |
| MMV000570 | 3 | 0 | 0 |
| MMV001230 | 3 | 0 | 0 |
| MMV001239 | 4 | 0 | 0 |
| MMV006389 | 4 | 0 | 0 |
| MMV007181 | 2 | 0 | 0 |
| MMV019017 | 4 | 0 | 0 |
| MMV306025 | 6 | 0 | 6 |
| MMV396736 | 6 | 0 | 5 |
| MMV403679 | 5 | 0 | 0 |
| MMV665794 | 5 | 0 | 0 |
| MMV665807 | 4 | 0 | 4 |
| MMV665852 | 4 | 0 | 4 |
| 27 compounds | **106** | **4** | **24** |

**Supplementary Table 2: *ScYRR1* mutations confer nonspecific drug resistance.** 106 total drug selections were performed on 27 compounds. Whole-genome sequencing of 106 resistant lines showed that seven of these (27%) had acquired mutations in *ScYRR1*, while only KAE609 resistant lines bore mutations in *ScPMA1.* All genomes were sequenced using the Illumina platform at 40-80x mean coverage.

| **Compound** |  | **ABC_16_-Monster modification** | | | |
| --- | --- | --- | --- | --- | --- |
| (uM) |  | *none* | *pma1L290S* | *yrrL611F* | *yrr1∆* |
| (+)**-**SJ000571311 ^2^ |  | 133.4±23.4 | 83.65±7.98 | ND | 93.9* |
| GNF-Pf4492^3^ |  | >150 | >150 | >150 | >150 |
| MMV020660^3,4^ |  | >150 | >150 | >150 | >150 |
| MMV666124^3,4^ |  | 72.9±23.3 | 87.6±48.3 | 56.24* | 71.65±24.9 |

**Supplementary Table 3. Activity of additional PfATP4 inhibitors against ABC_16_-Monster *S. cerevisiae*.** IC_50_’s were measured as described in methods and are the average of triplicate assays performed on two independent days. Error bars are standard error. ND—no data. * Single triplicate measurement.

| **Primer name** | **Sequence** |
| --- | --- |
| **414_VecF** | CCTTGACTAACTTATATGGTGCACTCTCAGTACAATCTGCTCTG |
| **414_GB_3R** | CGTGTAAGTTACAGGCAAGCGATCCTAAGAAACCATTATTATCATGACATTAACC |
| **414_GB_2F** | TTTCTTAGGATCGCTTGCCTGTAACTT |
| **414_GB_2R** | AAAGTGCCACCTGATCACGTGCTATAAAAATAA |
| **414_GB_1F** | TAATTATTTTTATAGCACGTGATCAGGTGGCACTTTTCGGGGAAATGTGC |
| **Pre-p426_Halfway_R** | CCCAACGATCAAGGCGAGTTAC |
| **414_MET15F** | CACACCGCATAGGCAAACAGCTTTACTAATCTTTACTTGTAATTTGGC |
| **New_414_MET15_R** | CAGATTGTACTGAGAGTGCACCATAAGTTAGTCAAGGCGCCATCCTC |
| **p426_Halfway_F** | GCGGCCAACTTACTTCTGAC |
| **New_414_Vec_Only_HalfR** | TGAGCTCCAGCTTTTGTTCC |
| **414_Vec_Half_F** | AATTAACCCTCACTAAAGGGAAC |
| **414_VecR** | CAAGTAAAGATTAGTAAAGCTGTTTGCCTATGCGGTGTGAAATA |
| **Attempt2_426_Vec_F** | GGAAGTACAGGACTATGGTGCACTCTCAGTACAATCTGC |
| **p426_Halfway_R** | GGTTAGCTCCTTCGGTCCTC |
| **p426_Halfway_F** | GCGGCCAACTTACTTCTGAC |
| **New_426_Vec_R** | CTCTGTCAGAAACGGCCTTAATTACCCTATGCGGTGTGAAA |
| **New_426_LEU2_F** | TTTCACACCGCATAGGGTAATTAAGGCCGTTTCTGACAGAG |
| **Attempt2_426_LEU2_F** | CTGAGAGTGCACCATAGTCCTGTACTTCCTTGTTCATGTG |

**Supplementary Table 4: CRISPR: Primers used to prepare the p414-TEF1p-Cas9-CYC1t-MET15 and p426-SNR52p-gRNA.CAN.Y-SUP4t-LEU2 plasmids.**

| **Primer name** | **Sequence** |
| --- | --- |
| PMA1-L290S-gRNA-fwd | tcCATTTCACTGAAGTTTtGAAgTTTTAGAGCTAGAAATAGCAAG |
| PMA1-L290S-gRNA-rev | aacTTCaAAACTTCAGTGAAATGgATCATTTATCTTTCACTGCGGAG |
| PMA1-P339T-gRNA-fwd | tcATAGTGGTGGTAACGACAGCgTTTTAGAGCTAGAAATAGCAAG |
| PMA1-P339T-gRNA-rev | aacGCTGTCGTTACCACCACTATgATCATTTATCTTTCACTGCGGAG |
| YRR1-L611F-gRNA-fwd | tcACAAAAAAACACcAATGTTTgTTTTAGAGCTAGAAATAGCAAG |
| YRR1-L611F-gRNA-rev | aacAAACATTgGTGTTTTTTTGTgATCATTTATCTTTCACTGCGGAG |
| YRR1-KO-gRNA-fwd | tcGCGGCGTAACGTTGGTGGCCgTTTTAGAGCTAGAAATAGCAAG |
| YRR1-KO-gRNA-rev | aacGGCCACCAACGTTACGCCGCgATCATTTATCTTTCACTGCGGAG |

**Supplementary Table 5: CRISPR: Primers used to prepare the gRNA plasmids.**

| **Primer Name** | **Sequence** |
| --- | --- |
| PMA1-L290S-Donor-UP | TTAACAAAGCCGCTGGTGGTCAAGGTCATTTCACTGAAGTTTcGAACGGTATTGGTATTATCTTATTGGTTTTGGTCATTGCCACTTTGT |
| PMA1-L290S-Donor-DOWN | ACAAAGTGGCAATGACCAAAACCAATAAGATAATACCAATACCGTTCgAAACTTCAGTGAAATGACCTTGACCACCAGCGGCTTTGTTAA |
| PMA1-P339T-Donor-UP | CACTCTAGGTATTACTATTATTGGTGTCCCAGTCGGTTTGaCAGCTGTCGTTACCACCACTATGGCCGTCGGTGCTGCTTACTTGGCTAA |
| PMA1-P339T-Donor-DOWN | TTAGCCAAGTAAGCAGCACCGACGGCCATAGTGGTGGTAACGACAGCTGtCAAACCGACTGGGACACCAATAATAGTAATACCTAGAGTG |
| YRR1-L611F-Donor-UP | GGCGTTATCAATGAGCCTCACGGCAGGGCTTTTTTCCAAAACATTcGTGTTTTTTTGTTCTCTGATCTACTTTAAGTTGAcGCTTTTCGA |
| YRR1-L611F-Donor-DOWN | TCGAAAAGCgTCAACTTAAAGTAGATCAGAGAACAAAAAAACACgAATGTTTTGGAAAAAAGCCCTGCCGTGAGGCTCATTGATAACGCC |
| YRR1-KO-Donor-UP | GGAAAGTTTATTGCCCTCAGCCGTGCCAATAAGAATAGCGTCACATGCTAAGTCGAGGCAATCTCGCGGCGCGACTACTTTGAAGCCTTT |
| YRR1-KO-Donor-DOWN | AAAGGCTTCAAAGTAGTCGCGCCGCGAGATTGCCTCGACTTAGCATGTGACGCTATTCTTATTGGCACGGCTGAGGGCAATAAACTTTCC |

**Supplementary Table 6: CRISPR: Oligonucleotides for DNA repair.**

| **Primer name** | **Sequence** |
| --- | --- |
| check-PMA1-fwd | CACTGTTAAGAGAGGTGAAGG |
| check-PMA1-rev | GTGTATGGTTCGTGCAAGG |
| check-YRR1-fwd | AGAGAAATTCGAAGCCTCC |
| check-YRR1-rev | GTCCTTTCAATAGCTAGCTC |
| check-YRR1-KO-fwd | ATGGGGTAGAGGCTGATATACG |
| check-YRR1-KO-rev | GGTAAGCAGCGATTCAGC |

##

## **Supplementary Table 7: CRISPR Primers for genomic-region PCR amplification and sequence verification.**

##

| **Name** | **Genotype** |
| --- | --- |
| SY025  (ABC_16_-Monster parent) | *MATa ho∆::[tetO2pr-GFP, URA3] can1∆::GMToolkit-a lyp1∆ his3∆1 leu2∆0 ura3∆0 met15∆0* |
| ABC_16_-Monster^5^ | *MATa adp1∆ snq2∆ ycf1∆ pdr15∆ yor1∆ vmr1∆ pdr11∆ nft1∆ bpt1∆ ybt1∆ ynr070w∆ yol075c∆ aus1∆ pdr5∆ pdr10∆ pdr12can1∆::GMToolkit-a lyp1∆ his3∆1 leu2∆0 ura3∆0 met15∆0* (deletions for the ABC transporter genes are marked with *[tetO2pr-GFP, URA3]*). |
| EAW122 | *MATa adp1∆ snq2∆ ycf1∆ pdr15∆ yor1∆ vmr1∆ pdr11∆ nft1∆ bpt1∆ ybt1∆ ynr070w∆ yol075c∆ aus1∆ pdr5∆ pdr10∆ pdr12can1∆::GMToolkit-a lyp1∆ his3∆1 leu2∆0 ura3∆0 met15∆0* (deletions for the ABC transporter genes are marked with *[tetO2pr-GFP, URA3]*),*pma1::L290S.* |
| EAW123 | *MATa adp1∆ snq2∆ ycf1∆ pdr15∆ yor1∆ vmr1∆ pdr11∆ nft1∆ bpt1∆ ybt1∆ ynr070w∆ yol075c∆ aus1∆ pdr5∆ pdr10∆ pdr12can1∆::GMToolkit-a lyp1∆ his3∆1 leu2∆0 ura3∆0 met15∆0* (deletions for the ABC transporter genes are marked with *[tetO2pr-GFP, URA3]*),*pma1::P339T.* |
| EAW100 | *MATa adp1∆ snq2∆ ycf1∆ pdr15∆ yor1∆ vmr1∆ pdr11∆ nft1∆ bpt1∆ ybt1∆ ynr070w∆ yol075c∆ aus1∆ pdr5∆ pdr10∆ pdr12can1∆::GMToolkit-a lyp1∆ his3∆1 leu2∆0 ura3∆0 met15∆0* (deletions for the ABC transporter genes are marked with *[tetO2pr-GFP, URA3]*), *yrr1∆.* |
| EAW125 | *MATa adp1∆ snq2∆ ycf1∆ pdr15∆ yor1∆ vmr1∆ pdr11∆ nft1∆ bpt1∆ ybt1∆ ynr070w∆ yol075c∆ aus1∆ pdr5∆ pdr10∆ pdr12can1∆::GMToolkit-a lyp1∆ his3∆1 leu2∆0 ura3∆0 met15∆0* (deletions for the ABC transporter genes are marked with *[tetO2pr-GFP, URA3]*), *yrrL611F.* |
| UCC4925 | *MATa/MATα his3Δ1/his3Δ1 leu2Δ0/leu2Δ0 ura3Δ0/ura3Δ0 lys2Δ0/+ trp1Δ63/+ hoΔ::P_SCW11_-cre-EBD78-NatMX/hoΔ::P_SCW11_-cre-EBD78-NatMX loxP-CDC20-Intron-loxP-HphMX/loxP-CDC20-Intron-loxP-HphMX loxP-UBC9-loxp-LEU2/loxP-UBC9-loxp-LEU2* |
| UCC9633 | *UCC4925 chrI(17068-17161)P_ADH1_-pHluorin-URA3/ chrI(17068-17161)P_ADH1_-pHluorin-URA3* |

**Supplemental Table 8:** **Yeast lines used in these experiments.**

## References

1 Meister, S. *et al.* Imaging of Plasmodium liver stages to drive next-generation antimalarial drug discovery. *Science* **334**, 1372-1377, doi:10.1126/science.1211936 (2011).

2 Jimenez-Diaz, M. B. *et al.* (+)-SJ733, a clinical candidate for malaria that acts through ATP4 to induce rapid host-mediated clearance of Plasmodium. *Proc. Natl. Acad. Sci. U. S. A.* **111**, E5455-E5462, doi:10.1073/pnas.1414221111 (2014).

3 Flannery, E. L. *et al.* Mutations in the P-Type Cation-Transporter ATPase 4, PfATP4, Mediate Resistance to Both Aminopyrazole and Spiroindolone Antimalarials. *ACS Chem. Biol.* **10**, 413-420, doi:10.1021/cb500616x (2015).

4 Lehane, A. M., Ridgway, M. C., Baker, E. & Kirk, K. Diverse chemotypes disrupt ion homeostasis in the malaria parasite. *Mol. Microbiol.* **94**, 327-339, doi:10.1111/mmi.12765 (2014).

5 Suzuki, Y. *et al.* The Green Monster Process for the Generation of Yeast Strains Carrying Multiple Gene Deletions. *Jove-J Vis Exp*, doi:UNSP e4072

DOI 10.3791/4072 (2012).
